# Damage thresholds for blaze diffraction gratings and grazing incidence optics at an X-ray free-electron laser

**DOI:** 10.1107/S1600577517016083

**Published:** 2018-01-01

**Authors:** Jacek Krzywinski, Raymond Conley, Stefan Moeller, Grzegorz Gwalt, Frank Siewert, Christoph Waberski, Thomas Zeschke, Daniele Cocco

**Affiliations:** a SLAC National Accelerator Laboratory, 2575 Sand Hill Road, Menlo Park, CA 94025, USA; b Argonne National Laboratory, 9700 South Cass Avenue, Argonne, IL 60439, USA; c Helmholtz Zentrum Berlin für Materialen und Energie, Albert-Einstein-Strasse 15, 12489 Berlin, Germany

**Keywords:** free-electron laser, optical damage, diffraction gratings

## Abstract

The Linac Coherent Light Source is upgrading its machine to high repetition rate and to extended ranges. Novel coatings, with limited surface oxidation, which are able to work at the carbon edge, are required. In addition, high-resolution soft X-ray monochromators become necessary. One of the big challenges is to design the mirror geometry and the grating profile to have high reflectivity (or efficiency) and at the same time survive the high peak energy of the free-electron laser pulses. For these reasons the experimental damage threshold, at 900 eV, of two platinum-coated gratings with different blazed angles has been investigated. The gratings were tested at 1° grazing incidence. To validate a model for which the damage threshold on the blaze grating can be estimated by calculating the damage threshold of a mirror with an angle of incidence identical to the angle of incidence on the grating plus the blaze angle, tests on Pt-coated substrates have also been performed. The results confirmed the prediction. Uncoated silicon, platinum and SiB_3_ (both deposited on a silicon substrate) were also investigated. In general, the measured damage threshold at grazing incidence is higher than that calculated under the assumption that there is no energy transport from the volume where the photons are absorbed. However, it was found that, for the case of the SiB_3_ coating, the grazing incidence condition did not increase the damage threshold, indicating that the energy transport away from the extinction volume is negligible.

## Abstract 2

The damage threshold of diffraction gratings and X-ray coatings have been studied using X-ray FEL radiation.

## 1. Introduction   

Free-electron lasers (FELs) operating in the extreme-ultraviolet to the X-ray regions are permitting unprecedented measurement techniques. Studies of the dynamics of chemical and physical phenomenon, diffraction imaging of non-periodic structures and the study of samples suffering radiation damage have become possible. In order to achieve all this, FEL pulses have very high peak power, ultrashort duration and are produced in a narrow photon bandwidth.

A new high-repetition-rate X-ray FEL, the Linear Coherent Light Source II (LCLS II), is under construction at SLAC National Laboratory. In order to fully utilize the new capabilities of LCLS II, new soft X-ray instruments are being developed. The performance of the instruments can become compromised by possible damage of the optical elements. In particular, grating monochromators, where the angle of incidence cannot be maintained very shallow over the entire range, have to be designed in a conservative way to prevent potential damage of the gratings. The main problem is that the FEL beam combines ultra-short pulse durations and high photon energies, which are usually much higher than the binding energies of solids. Damage studies campaigns, related to FELs, have been performed at various sources for single-shot and normal incidence conditions [see, for example, Hau-Riege *et al.* (2010 ▸), and references therein]. Recently, several damage studies at the grazing incidence condition in the hard X-ray regime were reported for single shots (Aquila *et al.*, 2015 ▸; Koyama *et al.*, 2013*a* ▸). There have been some multi-shot studies in the ultraviolet (Hau-Riege *et al.*, 2008 ▸) and extreme-ultraviolet or soft X-ray regimes (Juha *et al.*, 2009 ▸; Sobierajski *et al.*, 2016 ▸), which were carried out for normal incidence conditions and for non-metallic materials. However, investigations of damage due to multi-shot exposure of ultra-short intense X-ray pulses and at grazing incidence conditions have not been reported yet in the literature, except of our recent studies on platinum coating at 900 eV photon energy (Krzywinski *et al.*, 2015 ▸). Starting with the results obtained by Krzywinski *et al.* (2015 ▸), we have investigated other materials proposed for LCLS II and, more importantly for the beamline design, the damage threshold of blaze diffraction gratings.

## 2. Experimental   

### 2.1. Experimental setup   

The measurements were carried out at the SXR instrument at LCLS (Schlotter *et al.*, 2012 ▸). The experimental setup was the same as described by Krzywinski *et al.* (2015 ▸). The FEL beam produced in the undulator traverses the front-end enclosure (FEE) which houses the gas attenuator and the gas detectors measuring the pulse energy of the FEL beam for each pulse (Moeller *et al.*, 2011 ▸). A second gas detector (GMD) measures the average and shot-by-shot pulse energy (Tiedtke *et al.*, 2014 ▸) before reaching the sample. A Kirk­patrick–Baez (KB) refocusing mirror system (Kelez *et al.*, 2009 ▸) can change the beam size on the sample. The sample was mounted in the monitor tank downstream of the experimental chamber, which also houses a YAG screen to view the beam spot. The sample was mounted on a rotatable manipulator and was pre-aligned to the FEL beam coordinates to an accuracy of better than 0.1°. The LCLS photon energy was tuned to 900 eV. The FEL pulse energy was measured by the FEE gas detectors for each pulse. The transmission of the KB mirrors has been characterized previously to be around 50%. The beam size was monitored using a Ce:YAG sample mounted on the same sample holder. It was set to around 30 µm by using the bendable KB mirrors. The fluence was controlled by adjusting the gas attenuator pressure. During irradiation, the sample was monitored by using an Opal camera. The focused beam was characterized identically to our previous study (Krzywinski *et al.*, 2015 ▸) using an imprint method. The imprints have shown that the focused beam has a structure due to interference fringes caused by imperfection of the beamline optics. With the help of the imprints method we measured the, so-called, effective area *A*_eff_ (ISO 11254-1:2000; Chalupský *et al.*, 2010 ▸, 2013 ▸). The effective area is defined as *A*_eff_ = *E*_p_/*F*_max_, where *F*_max_ is the maximum fluence and *E*_p_ is the pulse energy and it is especially useful to define the peak fluence for non-Gaussian beams. The measured value of the effective area was 400 ± 50 µm^2^.

### 2.2. Samples   

The irradiated samples were: (1) X-ray optical quality silicon substrate, (2) 50 nm SiB_3_ layer deposited on silicon substrate, (3) platinum blazed grating with blaze angle of 0.7°, (4) platinum blazed grating with blaze angle of 1.4°. The bulk material for the gratings is single-crystal silicon wafer of 〈100〉 orientation. No binding layer or adhesive layer was used to increase the adhesion of the 30 nm-thick Pt or of the SiB_3_ to the silicon substrate. The grating samples have an unruled area that acts as a mirror and was also used for damage studies. The measured micro-roughness on all the samples was below 0.5 nm r.m.s., good enough to detect any damage induced on the optical surface by the radiation.

**Figure 1:**
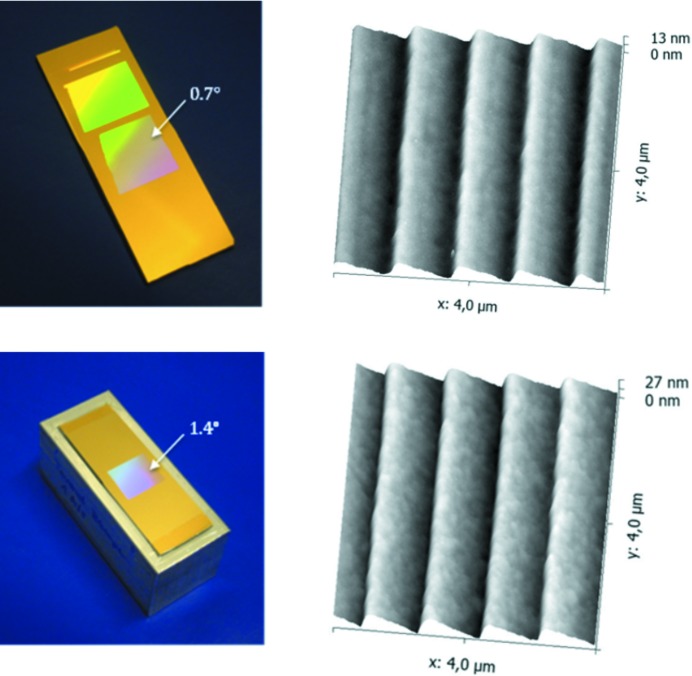
Photograph and AFM image of the 0.7° blazed grating sample (top) and the 1.4° blaze grating sample (bottom), after ruling into Au (photograph) and after etching and coating with Pt (AFM images).

The two blazed diffraction gratings (see Fig. 1 ▸) were produced at the grating laboratory of the Helmholtz Zentrum Berlin (HZB) (Siewert *et al.*, 2018 ▸). The gratings are mechanically ruled, using a Zeiss GTM-6 ruling machine (Loechel *et al.*, 2013 ▸). For this the silicon substrates were coated with gold as a ruling layer. A special diamond tool has been used to obtain a blazed grating pattern. This process defines the groove density of 1123 lines mm^−1^ but not the final blaze profile. Finally, the ruled pattern has been transferred to the silicon substrate by ion beam treatment (Nelles *et al.*, 2001 ▸). This process allows for excellent control of the desired blaze angle and in addition an improvement of the final micro-roughness on the grooves (Heidemann *et al.*, 2007 ▸). The blaze angles of the two gratings were chosen to be, in one case, identical to that used for the self-seeding monochromator at LCLS (Cocco *et al.*, 2013 ▸), and, in the other, to the future gratings for LCLS II. Fig. 1 ▸ shows the grating profiles as measured by use of an atomic force microscope (AFM) (Bruker SIS-Ultraobjective). On the 0.7° blazed grating the micro-roughness is 0.14 nm r.m.s. on the blaze side while 0.30–0.36 nm r.m.s. is found on the blaze side of the 1.4° grating sample.

The SiB_3_ was chosen because of its potential advantage as a soft X-ray coating. In fact, it was expected to have a higher damage threshold and lower oxidation state with respect to silicon. The carbon-free coating material can potentially be used across the entire LCLS II soft X-ray range (200–1600 eV). For all the expected grazing incidence angles of incidence, the reflectivity of SiB_3_ is expected to be above 85%. The coating was deposited on super polished substrates, at Argonne National Laboratory through the sputtering process. These measurements were supposed to be the first test for endorsing this coating as a viable solution for future soft X-ray FEL mirrors.

### 2.3. Irradiation conditions   

For the silicon and the SiB_3_ sample the grazing incidence angles were chosen to be 1.24° and for the platinum sample close to 2°. These conditions are similar to the expected operational conditions for mirrors and gratings, respectively. The exact values are presented in Table 1 ▸.

The grazing incident angles for grating samples were chosen such that the expected maximum absorbed dose was similar for the ruled and unruled area. The expected dose was simulated by solving the Helmholtz equation in non-homogeneous media as described by Krzywinski *et al.* (2015 ▸).

**Figure 2:**
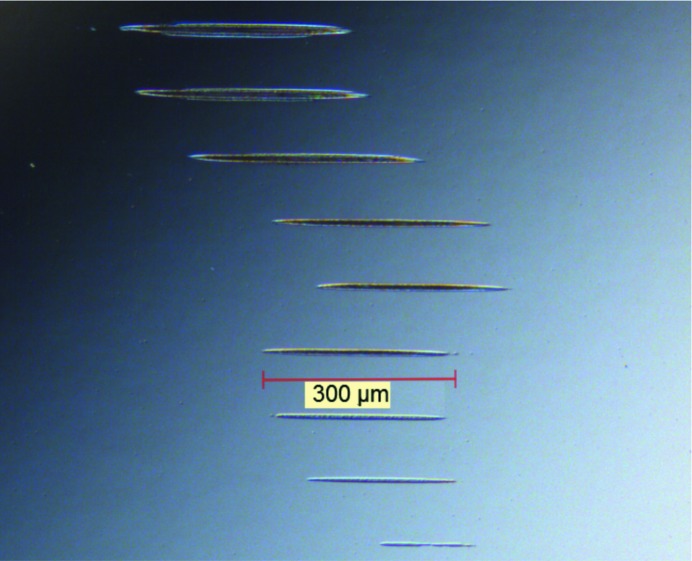
Examples of images showing damage of the Pt coating caused by 1000 shots of the focused X-ray beam (effective area 400 µm^2^) at a grazing incidence of 1.94°. Different images correspond to the beam transmission values in the range 0.13–1.4%. The image was taken using a Nomarski microscope.

We have performed single-shot and multi-shot irradiations for 1000 shots at different pulse energy levels (see Figs. 2 ▸, 3 ▸ and 4 ▸). The pulse energy was controlled by changing the gas pressure in the gas attenuator.

The average incoming FEL pulse energy, before the gas attenuator, was of the order of 1.6 mJ. We changed the transmission of the beamline, by changing the pressure of the gas, over the range 0.02–11%.

## 3. Data analysis and results   

The damage threshold at grazing incidence was determined as follows. First, the area of the damaged spots was plotted as a function of the attenuator pressure. The pressure is proportional to the logarithm of the transmission of the beamline. This plot relates to the so-called Liu plot (Liu, 1982 ▸), which is routinely used in damage threshold analysis. Second, we used linear regression and the last three points on the Liu plot to calculate the pressure *P*_th_ at which the damage vanishes. When the interpolated threshold was larger than the pressure at which we did not detect any damage, we assumed that *P*_th_ was in the range between the pressure that corresponded to the last visible spot and the pressure at which we did not detect any damage. The damage threshold is then determined as *F*_th_ = *E*_th_/*A*_eff_, where *E*_th_ is the transmitted energy corresponding to *P*_th_. The results are listed in Table 1 ▸. One can notice a striped pattern of the craters, which are caused by a non-Gaussian shape of the focused beam, which exhibit inhomogeneity due to interference fringes.

The maximum combined uncertainty in our measurement is about 35%. This is due to the individual uncertainties in the following: determining the attenuator pressure threshold values (0.5%), measuring the effective area (10%), determining the overall transmission (20%) and measuring the pulse energy (5%).

## 4. Discussion   

The main goal of this study was to compare damage thresholds of different coatings of grazing incidence optics. We have chosen multi-shot irradiations in order to be closer to operation conditions. Due to experimental constrains the maximum number of shots was limited to 1000. We have also performed some single-shot irradiations for Si and SiB_3_ samples. The Si sample does not show any cumulative effects whereas for the SiB_3_ case the single-shot damage is twice as high as for the multi-shot irradiations. This suggests different mechanisms for the cumulative effects for different materials. The mechanism of the cumulative effects is beyond the scope of this work and will be the subject of further studies.

A quantity that helps to assess the damage is the instantaneous absorbed dose per atom at the mirror surface (Krzywinski *et al.*, 2015 ▸),
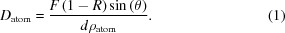
where *F* is the fluence, *R* is the reflectivity, 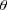 is the grazing incidence angle, 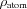 is the number of atoms per unit volume, 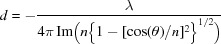
is the extinction length, λ is the wavelength and *n* is the complex refractive index.

The calculated absorbed doses for the damage thresholds of both 1000-shot irradiation at 120 Hz as well as single-shot irradiation are listed in Table 1 ▸. They vary between a fraction of an eV for SiB_3_ to a few eV for the platinum coating. The threshold absorbed dose for the platinum coating caused by 1000 shots agrees, within the experimental error, with the value measured for 600 shots published by Krzywinski *et al.* (2015 ▸). These dose values can be compared with the calculated energy density *D*_melt_ that is required to bring a solid to the melt temperature, which is a fraction of an eV. For example, in the case of platinum, *D*_melt_ ≃ 0.47 eV atom^−1^ (Koyama *et al.*, 2013*b* ▸). In general, the measured damage threshold at grazing incidence is higher than that calculated under the assumption that there is no energy transport from the volume where the photons are absorbed. This is attributed to the fact that a significant amount of absorbed energy is transported away from the surface before it melts (Aquila *et al.*, 2015 ▸; Krzywinski *et al.*, 2015 ▸; Liu, 1982 ▸). The SiB_3_ coating behaves very different from the Pt coating with respect to the energy deposition range. While in the case of the Pt coating the energy deposition range is much larger than the extinction length, the SiB_3_ energy deposition length is of the same order as the extinction length. One can speculate that this could be caused by much stronger electron–phonon coupling for SiB_3_ originating from drastically lower atomic weight and different band structure. The Si case is in between the Pt and SiB_3_ situation with the energy deposition length being only a few times longer than the extinction length. The experimental results presented in Table 1 ▸ confirmed that the absorbed dose was the same for the gratings and the flat mirrors when the incidence angles were set according to the theoretical predictions. In particular, for blazed gratings, it corresponds to the situation in which the angle of incidence on the mirror is identical to the angle of incidence on the facet of the grating groove, *e.g.* grazing angle of incidence plus the blaze angle.

The same conclusion was also derived from calculating the maximum absorbed dose by solving the Helmholz equation in non-homogeneous media, which was also successfully used to predict the damage threshold for laminar gratings (Gaudin *et al.*, 2012 ▸).

## 5. Summary   

Our results are important for the development of coated optics exposed to intense ultrashort X-ray pulses. They show that the effect of the grazing incidence illumination increases the damage threshold significantly beyond the limit predicted by theory, which is based on thermal equilibrium and the energy deposition mechanism due to photoabsorption. However, this effect depends on the composition of the coatings and for some materials, such as SiB_3_, the increase of the damage threshold is insignificant. Understanding the underlying processes that contribute to an increase of the damage threshold can help to design optics which can withstand higher instantaneous power and to optimize scientific instruments at XFELs. One important finding of this research is that the blaze gratings, from the point of view of the damage threshold, can be treated as mirrors when irradiated at an angle identical to the angle between the beam and the grating facets, *e.g.* the incident angle on the grating plus the blaze angle of the grooves. This basic rule simplifies considerably the calculation of the safety condition for gratings under FEL radiation.

## Figures and Tables

**Figure 3:**
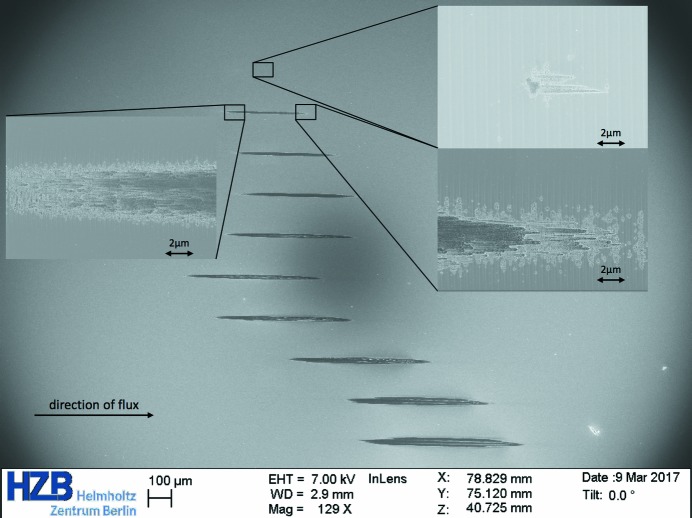
Examples of images showing the damage of the 0.7° blazed grating Pt-coated sample caused by 1000 shots of the focused X-ray beam (effective area 400 µm^2^) at a grazing incidence of 1.24°. The images were taken with a Zeiss LEO 1560 scanning electron microscope. Different imprints correspond to beam transmission values in the range 0.13–1.4%.

**Figure 4:**
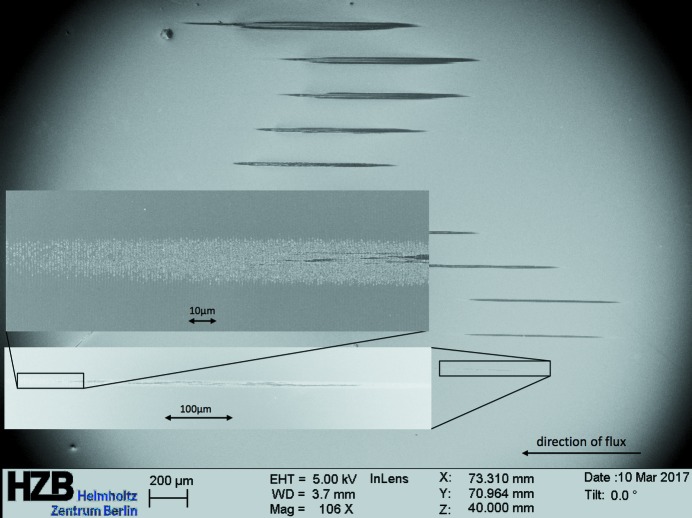
Examples of images showing the damage of the 1.4° blazed grating Pt-coated sample caused by 1000 shots of the focused X-ray beam (effective area 400 µm^2^) at a grazing incidence of 0.94°. The images were taken with a Zeiss LEO 1560 scanning electron microscope. Different imprints correspond to beam transmission values in the range 0.13–1.4%.

**Table 1:** Damage thresholds determined for different irradiation conditions The maximum absorbed dose (right column) was calculated from the measured fluence damage thresholds using equation (1).

Sample	Irradiation type	Angle of incidence (°)	Damage threshold fluence (J cm^−2^)	Maximum absorbed dose (eV atom^−1^)
Pt mirror	1000 shots	2.34	0.35 ± 0.12	3.7
Pt grating 1.4°	1000 shots	0.94	0.35 ± 0.12	3.7
Pt mirror	1000 shots	1.94	0.42 ± 0.15	3.8
Pt grating 0.7°	1000 shots	1.24	0.42 ± 0.15	3.8
Si	1000 shots	1.24	1.4 ± 0.5	1.7
Si	Single shot	1.24	1.4 ± 0.5	1.7
SiB_3_	1000 shots	1.24	0.35 ± 0.12	0.2
SiB_3_	Single shot	1.24	0.42 ± 0.15	0.4
